# Antiproliferative effects of dried *Moringa oleifera* leaf extract on human Wharton’s Jelly mesenchymal stem cells

**DOI:** 10.1371/journal.pone.0274814

**Published:** 2022-10-05

**Authors:** Kivaandra Dayaa Rao Ramarao, Chandran Somasundram, Zuliana Razali, Wijenthiran Kunasekaran, Tan Li Jin, Sabri Musa, Vijayan Manickam Achari

**Affiliations:** 1 Institute of Biological Sciences, Faculty of Science and The Centre for Research in Biotechnology for Agriculture (CEBAR), University of Malaya, Kuala Lumpur, Malaysia; 2 Cytonex Sdn. Bhd., Menara UOA Bangsar, Bangsar, Kuala Lumpur, Malaysia; 3 Department of Paediatric Dentistry & Orthodontics, Faculty of Dentistry, University of Malaya, Kuala Lumpur, Malaysia; 4 Institute of Biological Sciences, Faculty of Science, University of Malaya, Kuala Lumpur, Malaysia; University of Windsor, CANADA

## Abstract

Mesenchymal stem cells (MSCs) have seen an elevated use in clinical works like regenerative medicine. Its potential therapeutic properties increases when used in tandem with complementary agents like bio-based materials. Therefore, the present study is the first to investigate the cytotoxicity of a highly valued medicinal plant, *Moringa oleifera*, on human Wharton’s Jelly mesenchymal stem cells (hWJMSCs) and its effects on the cells’ gene expression when used as a pre-treatment agent *in vitro*. *M*. *oleifera* leaves (MOL) were dried and subjected to UHPLC-QTOF/MS analysis, revealing several major compounds like apigenin, kaempferol, and quercetin in the MOL, with various biological activities like antioxidant and anti-cancer properties. We then treated the hWJMSCs with MOL and noticed a dose-dependant inhibition on the cells’ proliferation. RNA-sequencing was performed to explain the possible mechanism of action and revealed genes like PPP1R1C, SULT2B1, CDKN1A, mir-154 and CCNB1, whose expression patterns were closely associated with the negative cell cycle regulation and cell cycle arrest process. This is also evident from gene set enrichment analysis where the GO and KEGG terms for down-regulated pathways were closely related to the cell cycle regulation. The Ingenuity pathway analysis (IPA) software further predicted the significant activation of (p < 0.05, z-score > 2) of the G2/M DNA damage checkpoint regulation pathway. The present study suggests that MOL exhibits an antiproliferative effect on hWJMSCs via cell cycle arrest and apoptotic pathways. We believe that this study provides an important baseline reference for future works involving MOL’s potential to accompany MSCs for clinical works. Future works can take advantage of the cell’s strong anti-cancer gene expression found in this study, and evaluate our MOL treatment on various cancer cell lines.

## Introduction

Plants have been treated as important reservoirs of essential nutrients and phytochemicals used for centuries to treat diseases. Most consumers utilise plant-derived products due to their long-established dietary patterns and medicinal properties. Recent trends have shown that most conventional drugs are sourced or derived from plants [1]. One of the best-known medicinal plants is *Moringa oleifera*, also called the ’drumstick’ tree and ’Mother’s best friend’ [2]. *M*. *oleifera* leaves (MOL) are traditionally utilised as a food source, where it is added to soups and curries [3]. The powdered preparation of dried MOL has increased popularity as an antioxidant supplement because of the wide range of antioxidant compounds such as phenolic acids, flavonoids, and vitamin E [4, 5]. The medicinal properties of most plants have been primarily attributed to these antioxidant compounds. In MOL, these compounds have been attributed to confer its pharmacological properties like anti-cancer, anti-inflammatory, anti-diabetic, hepatoprotective, and anti-hypertensive activity [6, 7]. Compounds like quercetin, apigenin, kaempferol and many others can induce apoptosis and downregulate the proliferation of cells [8, 9]. Moringin, a major glucosinolate compound found in *M*. *oleifera* could induce cell cycle arrest of SH-SY5Y cells through the involvement of pro-apoptotic factors like p21, and p53 [10]. When treated with MOL extract, multiple proliferation-related factors like Akt, NF-kB, p-Erk, β-catenin, and cyclin D1 were significantly downregulated in A549 lung cancer cells [11, 12].

Plants have been extensively studied for their ability to regulate transcriptional mechanisms and biological processes, especially for the in-depth understanding of basic stem cell or cancer cell biology [13]. Mesenchymal stem cells (MSCs) are of particular interest due to their vast potential in clinical works like regenerative therapy. Whilst most are used alone in registered clinical trials, some have purported that its application with complementary agents like plant extracts or biomaterials can enhance its therapeutic efficiency. MSCs can be sourced from multiple regions in the human body, such as bone marrow and adipose tissue. The human Wharton’s Jelly mesenchymal stem cells (hWJMSCs) derived from the umbilical cord has recently seen elevated clinical use due to benefits like simple isolation, high proliferation, and broad differentiation potential [14]. Furthermore, the isolation of hWJMSCs is non-invasive and thus circumvents any ethical concerns because the umbilical cord is considered medical waste.

So far, it remains elusive whether MOL affects the biology of hWJMSCs. While researchers have identified many plants with high antioxidant properties, few have been evaluated for their effects or potential as pre-treatment agents for MSCs, potentially enhancing the cells’ therapeutic efficiency depending on their reaction to the treatment. Therefore, this study was the first (to the best of our knowledge) to take advantage of MOL’s known high antioxidant properties and investigate its cytotoxicity on hWJ-MSCs. RNA-seq analysis was also conducted to evaluate changes in the gene expression profile of the MOL-treated hWJMSCs compared to untreated hWJMSCs. The outcome of our study can help us attempt to draw up recommendations for the use of MOL as a potential candidate to enhance the clinical efficiency of hWJMSCs.

## Materials and methods

### Sample preparation

MOL (250 g) was purchased from a local market in Petaling Jaya, Malaysia, located 6 km away from the Postharvest Laboratory, University of Malaya, Malaysia. Mature leaves, dark and fully expanded [15] were individually separated by hand. Excess leaves that did not meet these criteria were discarded. The separated leaves were then weighed (10 g) and subjected to drying in a convection-air based drying system at 60°C and drying air velocity of 1.7 ms^-1^ (Pre-optimised drying temperature and air velocity for MOL using the dryer for highest antioxidant properties). Details on the dryer have been described in a previous study [16]. MOL was dried until constant weight, and the corresponding moisture content was 5% (wet basis). The dried leaves were then ground into powder under liquid nitrogen using pestle and mortar. The resulting powdered sample was immediately extracted [17] where a mixture of sample and 80% methanol in a ratio of 1:4 was agitated at 200 rpm for 30 min at room temperature using an orbital shaker (Shellab, USA), then centrifuged (Beckman, USA) at 6500 rpm for 10 minutes at 4°C. The supernatant was collected and stored at– 20°C until subsequent analysis.

### Ultra-High-Performance liquid chromatography (UHPLC-QToF/MS) analysis

The supernatant described above was concentrated in a rotary evaporator, and the analysis was carried out using the resulting dried MOL extract [6, 18]. The dried extract was re-suspended in methanol (1 mg/mL) and filtered with a 0.22 μm membrane before injection into the UHPLC system. Acetonitrile and methanol were UHPLC-MS grade (Fisher Scientific, Belgium), while formic acid (MS grade) was from Sigma-Aldrich (St. Louis, USA). The analysis was performed on an ACQUITY UPLC I-Class system (Waters, USA). The column used was the ACQUITY UPLC HSS T3 (100 mm x 2.1 mm x 1.8 μm), also from Waters. A linear binary gradient of water (0.1% formic acid) and acetonitrile were used as mobile phases A and B, respectively. The mobile phase composition during the run was: 0 min, 1% B; 0.5 min, 1% B; 16.00 min, 35% B; 18.00 min, 100% B; 20.00 min, 1% B. The flow rate was set to 0.6 mL/min, and the injection volume was 1 μL. The UHPLC system was coupled to a Vion IMS QTOF hybrid mass spectrometer (Waters). The ion source was operated in negative electrospray ionisation (ESI) mode under the following conditions: capillary voltage, 1.50 kV; reference capillary voltage, 3.00 kV; source temperature, 120°C; desolvation gas temperature, 550°C; desolvation gas flow, 800 L/h, and cone gas flow, 50 L/h. Nitrogen (>99.5%) was employed as the desolvation gas. Data were acquired in HDMS^E^ (High Definition MS^E^) mode in the range of m/z 50–1500 at 0.1 s/scan. Two independent scans were alternatively acquired: a low-energy scan at a fixed collision energy of 4 eV and a high-energy scan where the collision energy was ramped from 10 to 40 eV. Argon (99.999%) was used as collision-induced-dissociation (CID) gas.

### Cell culture work

#### Preparation of extract

The dried MOL extract was dissolved in Dulbecco’s phosphate-buffered saline (dPBS) for 10 mg/ml stock solution. Extracts were kept at −20°C until further analyses.

#### Culturing mesenchymal stem cells (MSCs)

Passage 2 (P2) Wharton’s jelly mesenchymal stem cells (WJ-MSCs) were obtained from Cytonex Sdn. Bhd. The donor voluntarily consented to participate in the study by signing the consent form in the presence of a doctor. The signed consent forms were documented in Cytonex Sdn Bhd. The Medical Ethics Committee, Faculty of Dentistry, the University of Malaya granted ethics approval for collecting and using the cells [DF CD1411/0087(P)]. Briefly, the preparation steps were conducted by Cytonex Sdn. Bhd. as follows: Once the cord was received, it was washed twice in sterile ultrapure water and transferred into a tube filled with Dulbecco’s phosphate-buffered saline without calcium and magnesium [DPBS (-,-)]. Arteries from the cord were removed and subjected to enzymatic digestion (Collagenase Type I, 3 mg/ml) and mechanical digestion (mincing). The tissues were then incubated at 37°C, 5% CO_2_ for 24 hours. The tissues were then cultured in complete cell culture media, made up of KO-DMEM (GibcoBRL, USA) supplemented with 10% fetal bovine serum (Invitrogen, USA), 1% Glutamax and 0.25% gentamicin (GibcoBRL). Cells were maintained up to passage 2 (P2), which were then used in subsequent experiments.

### hWJMSC characterisation study

#### Immunophenotyping

Immunophenotyping was conducted using BD Stemflow TM Human MSC Analysis Kit (BD Biosciences, San Jose, USA). Cells were seeded in T-75 flasks at a density of 1500 cells/cm^2^ for 48 h. Upon culture medium removal, the cells were washed with 1X dPBS and detached with trypsin (TrypLE™ Express Enzyme (1X), GibcoBRL, USA). Antibodies against MSC markers were added according to the manufacturer’s instructions. Briefly, a mixture of three antibodies (conjugated with different fluorophores) was used to recognise positive surface markers: CD90-FITC, CD105-PerCP-CyTM 5.5 and CD73-APC of PE-labeled antibodies that recognise the negative surface markers: CD45, CD34, CD11b, CD19, and HLA-DR. For compensation, CD90-FITC, CD44-PE, CD105-PerCP-Cy^TM^ 5.5, and CD73-APC were used. Antibody/cell mixtures were incubated in the dark on ice for 30 minutes, then centrifuged at 1500 rpm for 5 minutes, washed twice with 100 μL stain buffer, and re-suspended in 500 μL stain buffer (BD Pharmingen stain buffer, BD Biosciences, USA). The analysis was conducted with a BD FACSVia flow cytometer.

#### Trilineage differentiation

Cells were seeded into 6-well plates at a density of 1500 cells/cm^2^ and kept at 37°C, 5% CO_2_. A third of the cell culture medium was replaced every 3 days. For osteogenic differentiation, StemPro® Osteogenesis Differentiation Kit (Gibco, USA) replaced the cell culture medium, and calcium deposits were visualised using Alizarin Red S (Sigma, USA) after 21 days. For chondrogenic differentiation, StemPro® Chondrogenesis Differentiation Kit (Gibco, USA) was used, and chondrocytes were visualised using Alcian blue (Sigma, USA) after 14 days. Adipogenic differentiation was conducted using StemPro® Adipogenesis Differentiation Kit (Gibco, USA), and lipid droplets were visualised using Oil Red O (Sigma, USA) after 14 days.

### Cytotoxicity study of MOL on hWJMSCs

#### Cell viability test

Cells were seeded into a 96-well plate containing a cell culture medium at a density of 1500 cells/cm^2^. Control samples were incubated for 48 h in 5% CO_2_, 37°C incubators. For the treated samples, MOL extract was added to the wells with final concentrations of 100, 200, 400, 600, 800 and 1000 μg/ml and similarly left to incubate at 37°C for 48 h [19–22]. After 48 h, 70 μL of XTT reagent was added to each well and further incubated at 37°C for 4 hours. Then, XTT-specific absorbance was measured using the formula:

Specific Absorbance = [Abs_450nm_ (Test)–Abs_450nm_(Blank)]–Abs_660nm_(Test)

The specific absorbance signal was compared between treated cells and untreated cells to determine viable cells per well. The IC_50_ and IC_20_ concentrations were determined [23]. The values were interpolated from a cubic spline dose-response curve using GraphPad Prism 6.0 (GraphPad Software, San Diego, USA). The MOL extract’s IC_20_ concentration (non-cytotoxic dose) was used for subsequent work, including population doubling time, morphology analysis, and gene expression patterns in the hWJMSCs treated with our MOL extract [24].

#### Population doubling time (PDT)

PDT of cells stimulated with the IC_20_ MOL extract was analysed within 144 h using the formula:

$$PDT=t\times [lg2/(lgN_{t}-lgN_{0})]$$

where t is the culture time (h), N_t_ is the harvested cell number, N_0_ is the initial cell culture number.

#### Cell morphology

Cell morphology analysis was conducted concurrently with PDT analysis from 0 h– 144 h using an inverted microscope inverted phase-contrast microscope (Olympus CKX53, Japan) after adding the IC_20_ MOL extract.

### Gene expression analysis in hWJMSCs treated with MOL

#### RNA-sequencing protocol

RNA sequencing was conducted by Apical Scientific Sd. Bhd. (commercial lab) using the poly(A)-seq library generation and sequencing method [25] with slight modifications. Total RNA was extracted with RNeasy Mini Kit, and the purity and integrity of the RNA were determined by NanoPhotometer® spectrophotometer (IMPLEN, USA) and Agilent 2100 bioanalyser (Agilent, USA), respectively. Library preparation involved the use of 1 μg RNA for control and treated samples each. Sequencing libraries were generated using NEBNext® Ultra TM RNA Library Prep Kit for Illumina® (NEB, USA) following the manufacturer’s instructions. The mRNA in our samples were purified using poly-T oligo-attached magnetic beads. The first cDNA strand was synthesised using random hexamer primer and M-MuLV Reverse Transcriptase (RNase H). In contrast, second-strand cDNA was subsequently synthesised using DNA Polymerase I and RNase H. After the 3’ ends adenylation, NEBNext Adaptors were ligated to the synthesised strands for hybridisation. cDNA fragments of 150~200 bp in length were purified with the AMPure XP system (Beckman Coulter, USA). Then 3 μl USER Enzyme (NEB, USA) was added to the adaptor-ligated cDNA at 37°C for 15 min followed by 5 min at 95°C before PCR. Subsequently, PCR was performed with Phusion High-Fidelity DNA polymerase, Universal PCR primers and Index (X) Primer, followed by purification of the products using the AMPure XP system. Then, sequencing was performed using the Illumina NovaSeq 150 PE platform according to the manufacturer’s instructions. Quality control was performed to obtain clean reads by removing raw reads with adapter contamination and poly-N sequences. The reference genome "homo sapiens UCSC hg19" and gene model annotation files were retrieved from genome websites (NCBI/UCSC/Ensembl). Paired-end reads were then aligned to the reference genome using the Spliced Transcripts Alignment to a Reference (STAR) software against the reference genome. The relative abundance of transcripts was normalised using the FPKM (fragment per kilobase of exon per million fragmented mapped) strategy: (1000 x read count)/(number of gene covered bases x number of mapped fragments in a million) [26]. Differential gene expression analysis was performed using the edgeR R package, where significant differentially expressed genes (DEGs) were selected based on the threshold of log_2_ fold change > 1 and p-value < 0.05 by comparing control and MOL treated groups [27].

### Gene set enrichment analysis (GSEA) and Ingenuity Pathway Analysis (IPA)

GSEA of RNA-sequencing data was performed using the pre-ranked method [28] with slight modifications. The differentially expressed genes between the treated and control samples were ranked according to their log_2_ fold change values. The pre-ranked gene list was then loaded for analysis. The number of permutations was set to 1000 as default. All gene sets analysed in this study were from the Molecular Signatures Database (MSigDB). A positive normalised enrichment score (NES) represents a gene set that was up-regulated and vice versa. GO and KEGG terms with p < 0.05 were considered significantly enriched. Functional analyses to predict networks affected by the differentially expressed genes were also carried out using Ingenuity Pathways Analysis (IPA) software (Ingenuity^®^ Systems, http://www.ingenuity.com/). The previously identified DEGs (log2 fold change > 1 and p-value < 0.05) were uploaded into the IPA system for core analysis to identify canonical pathways, upstream regulators, diseases, and functions. IPA predicts pathways based on scores generated from a hypergeometric distribution, where the significance level as defined by the user is obtained by Fisher’s exact test at the right tail [29]. In order to not limit the analysis only with the *in vitro* model, IPA filter settings were not restricted to mesenchymal cells [30]. For canonical pathway analysis, upstream regulator analysis and disease and function analysis, the −log (p-value) > 1.3 was chosen as a threshold, whilst a z-score > 2 was used to depict the threshold of significant activation, and z-score < −2 for significant inhibition [29]. The z-score calculation algorithm used by IPA has already been described previously [31].

## Results

### UHPLC-QTOF/MS analysis

UHPLC is an improved form of liquid chromatography with a significant reduction in retention times, and subsequently, reduced time of analysis and reduced solvent consumption compared to the high-performance liquid chromatography (HPLC) method [32]. In this study, formic acid and acetonitrile were employed as the mobile solvents, where both are common mobile phases developed for phenolic determination [33]. In *M*. *oleifera*, a mobile phase of acetonitrile and 0.1% formic acid aqueous solution was previously found to produce peaks with superior symmetry and good ionisation of analytes [34]. Furthermore, we employed the negative electrospray ionisation (ESI) mode because it employs higher collision energy, producing adequate fragmentation for the analyte’s determination [35]. A recent study on *M*. *oleifera* found that the ESI’s negative mode was better than the positive mode based on the quantity and the responses of the identified compounds [6]. The top 20 compounds identified using the UHPLC-QTOF/MS method in our dried MOL extracted are shown in Table 1 below. Based on Table 1, the top compounds identified in our methanolic MOL extract includes Apigenin-6-C-glucosylglucoside, Quercetin-3-O-(6"-O-acetyl)-β-D-glucopyranoside, Cimicifugic acid A and Chlorogenic acid. Flavonoids are diverse in glycosylation sites, with substitutions mainly as O- and C-glycosides, giving rise to various derivatives [36]. Apigenin is one such example where the presence of a C-glycoside has been shown to stabilise its antioxidation performance whilst eliminating its pro-oxidant effect [37]. Among those compounds shown in Table 1, almost all have known biological activities such as antioxidative or anti-tumour, where 40% are flavonoid derivatives, and the rest comprise phenolic acids, sugars, lignans, anthraquinones, stilbenoids, quinnic acid, sesquiterpenes and organic acids as reported previously in *M*. *oleifera* leaf extracts [4, 6].

**Table 1:** The top 20 phytochemicals identified in MOL using the UHPLC-QTOF/MS method.

Component name	Formula	Neutral mass (Da)	Observed neutral mass (Da)	Observed m/z	Mass error (ppm)	Observed RT (min)	Observed CCS (Å^2^)	Intensity (%)
Apigenin-6-C-glucosylglucoside	C_27_H_30_O_15_	594.15847	594.1589	593.1516	0.7	6.9	241.09	12.26
Quercetin-3-O-(6"-O-acetyl)-β-D-glucopyranoside	C_23_H_22_O_13_	506.10604	506.1063	505.099	0.5	9.54	214.77	7.08
Cimicifugic acid A	C_21_H_20_O_11_	448.10056	448.1006	447.0933	0	9.92	203.69	5.64
Chlorogenic acid	C_16_H_18_O_9_	354.09508	354.0951	353.0879	0.2	3.94	173.81	5.15
Suffruticoside D	C_27_H_32_O_16_	612.16903	612.1689	611.1616	-0.2	5.59	232.93	4.93
Aloeemodin-8-monoglucoside	C_21_H_20_O_10_	432.10565	432.1058	431.0985	0.3	8.54	197.59	1.92
Eriodictyol-7-O-β-D-methyl-glucuronopyranoside	C_22_H_22_O_12_	478.11113	478.1109	477.1036	-0.4	10.23	211.63	0.69
4-Feruloylquinic acid	C_17_H_20_O_9_	368.11073	368.1109	367.1036	0.5	5.57	182.07	0.58
α-Kojibiose	C_12_H_22_O_11_	342.11621	342.1163	341.109	0.3	0.58	175.61	0.45
Sophorabioside	C_27_H_30_O_14_	578.16356	578.1631	577.1558	-0.8	8.09	237.67	0.44
Kaempferol-3-O-β-D-glucopyranoside	C_21_H_20_O_11_	448.10056	448.1008	447.0935	0.4	7.79	207.49	0.41
Sanleng acid	C_18_H_34_O_5_	330.24062	330.2413	329.2341	2.2	16.09	189.67	0.40
Kaempferol 3-α-L-dirhamnosyl-(1→4)-β-D-glucopyranoside	C_27_H_30_O_15_	594.15847	594.1589	593.1517	0.8	7.48	244.47	0.37
Indigoticoside A	C_26_H_34_O_11_	522.21011	522.2102	521.2029	0.2	9.55	223.66	0.36
Shancilin	C_30_H_28_O_6_	484.18859	484.1883	483.181	-0.6	15.67	217.79	0.35
Smiglanin	C_15_H_16_O_9_	340.07943	340.0797	339.0724	0.7	4.49	176.31	0.30
Pterodontoside F	C_21_H_38_O_8_	418.25667	418.2568	417.2495	0.3	11.27	212.69	0.28
Quinic acid	C_7_H_12_O_6_	192.06339	192.0637	191.0564	1.5	3.73	175.18	0.27
Eriocitrin	C_27_H_32_O_15_	596.17412	596.1742	595.167	0.2	6.04	243.67	0.19
Isorhamnetin 3-O-rhamnoside	C_22_H_22_O_11_	462.11621	462.1164	461.1091	0.4	10.6	219.97	0.16

*RT = Retention time

*CSS = Collision cross section

### MSC characterisation analysis

#### Immunophenotyping

**Fig 1:**
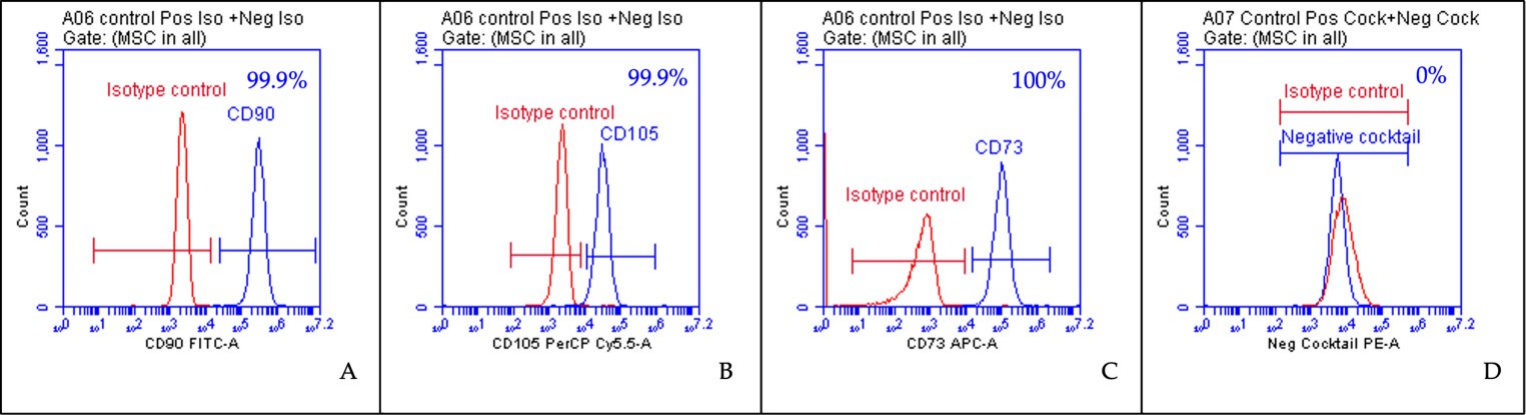
**Flow cytometry histogram of hWJ-MSCs for MSC positive markers (CD90, CD105, CD44) and negative markers (CD45, CD34, CD11b, CD19, and HLA-DR).** The blue histogram indicates background fluorescence obtained with the specific antibodies, while the red represents the isotype control.

The immunophenotype of the hWJ-MSCs was detected using flow cytometry, and the results are shown in Fig 1. The results revealed that the hWJ-MSCs used in this study highly expressed the positive surface markers, ranging from 99.9 to 100%. The expression of negative markers were 0%. This fulfils the criteria set by The International Society for Cellular Therapy (ISCT) regarding the definition of human MSCs where ≥95% of the MSC population must express CD105, CD73 and CD90, and lack expression (≤2% positive) of CD45, CD34, CD11b, CD19 and HLA-DR [38].

#### Trilineage differentiation

**Fig 2:**
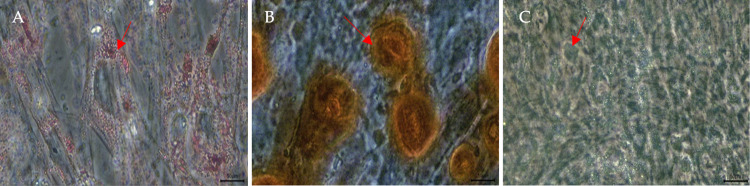
Appearance of adipocytes (A), osteocytes (B) and chondrogenic (C) differentiation of WJ-MSCs (Magnification, x4; scale bar = 50 μm).

The cells were further subjected to the trilineage differentiation experiment to verify their status as MSCs. Based on Fig 2A, the Oil Red O stain highlighted a red-orange colour, indicating the presence of lipid-rich intracytoplasmic vacuoles after adipogenic differentiation. Calcium deposits were apparent after osteogenic differentiation, evident from the reddish stains observed (Fig 2B). Chondrogenic differentiation of chondro was also confirmed by Alcian blue staining, where the blue sports indicates the presence of functional chondrocytes (Fig 2C). Consequently, trilineage differentiation of the cells used in this study was achieved, confirming its identity as MSCs.

### MOL cytotoxicity analysis

#### Effects of MOL on hWJMSCs viability

**Fig 3:**
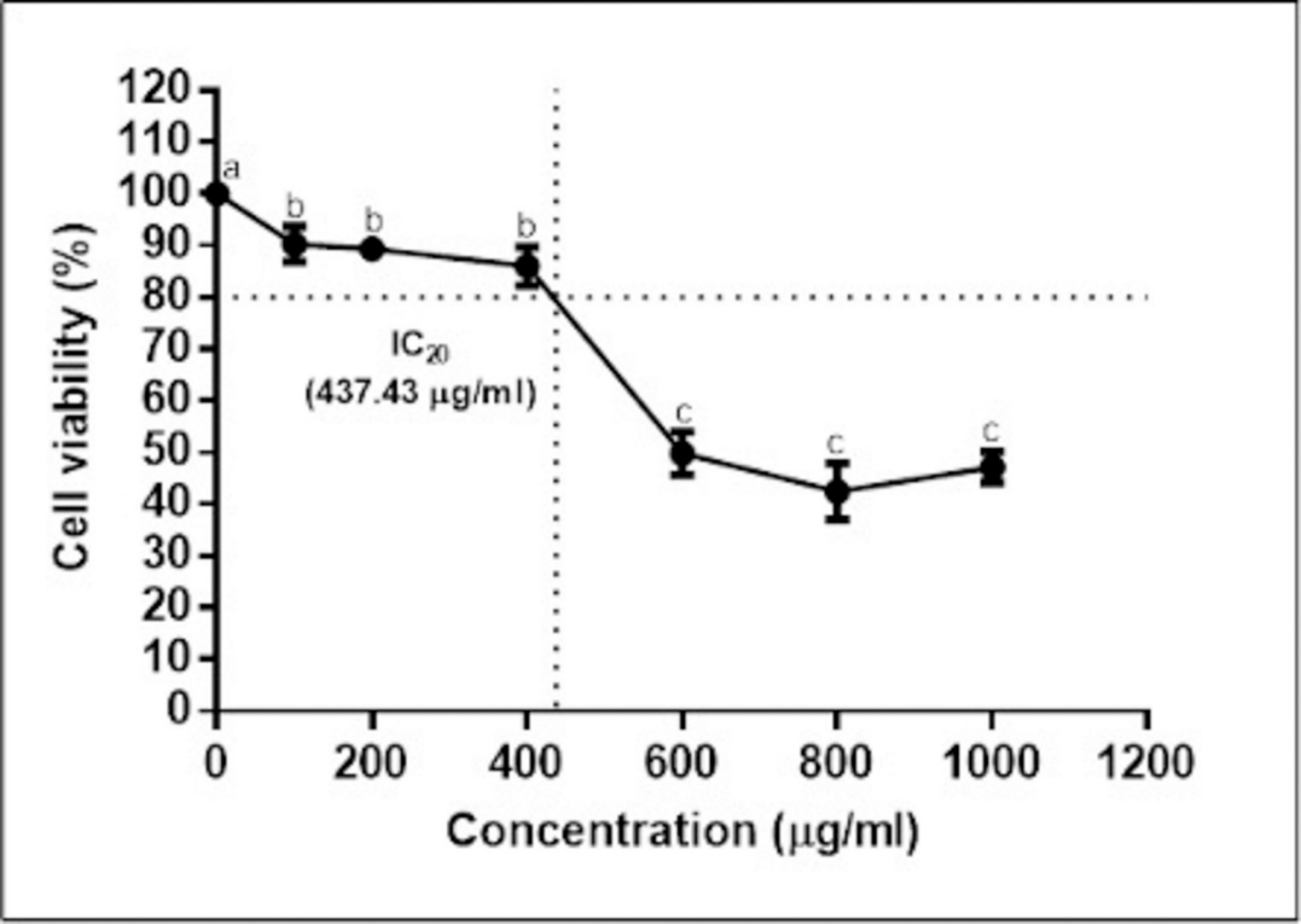
Effects of different concentrations of MOL on the cell viability (%) of hWJ-MSCs after 48 h. The results were reported as mean ± SD. Significant difference (p < 0.05) among treatments were represented by different superscripts.

A major concern relating to using plant extracts as complementary agents to cell-related work is their toxicity. Therefore, a cell viability test was conducted using the XTT assay to evaluate the cytotoxicity of MOL on hWJ-MSCs. Control and treated cells were exposed to various concentrations of MOL for 48 h and the results are shown in Fig 3. We noticed a dose-dependent inhibition of the leaf extract on the MSCs and that the IC_20_ value was 437.43 μg/ml.

The IC_20_ concentration was used for subsequent analysis. In cytotoxicity studies, the IC_20_ value is considered an important parameter where it is referred to as the non-cytotoxic dose. This is important in studying the effects of chosen compounds or drugs, especially for gene expression studies [39]. The IC_50_ concentration was also determined at 610.55 ± 41.38 μg/ml, and this showed that the MOL in this study is considered non-toxic, where according to US National Cancer Institute for plant compound screening, only IC_50_ values ≤ 20 μg/ml falls under the very toxic category [40].

#### Morphological analysis

**Fig 4:**
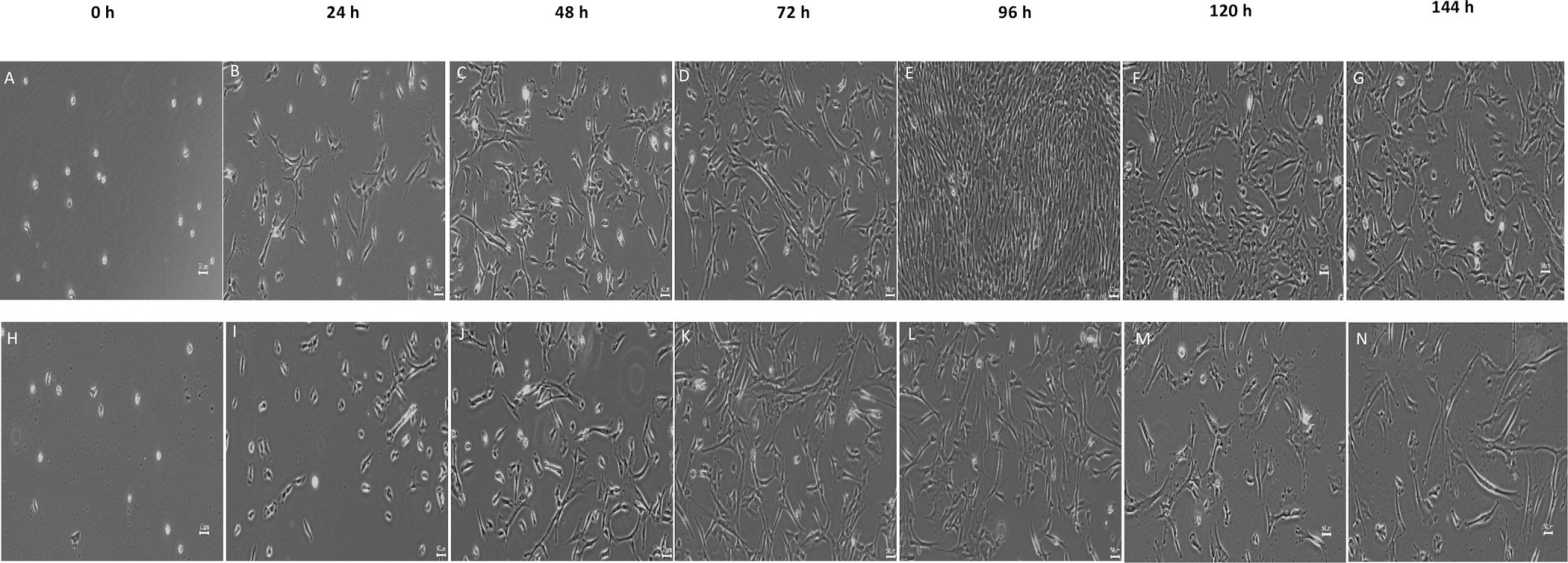
Morphology of untreated (A-G) and MOL treated (H-N) hWJ-MSCs from 0 h to 144 h (Magnification, x4; scale bar = 50 μm).

The morphology of hWJ-MSCs treated with MOL was analysed under a microscope, and the results are presented in Fig 4. The cell morphology changed over the culture period, where it started as small, rounded cells that have yet to attach to the culture plate at 0 h (Fig 4A and 4H). This was followed by flattened, fibroblastic-like, spindle-shaped cells from 24–144 h. Both cell populations proliferated quickly up until 72 h, after which untreated cells (control) continued growing to 90–100% confluence until 96 h (Fig 4E), followed by a decrease in cell number (Fig 4F and 4G) from 120–144 h, whereas MOL treated cells dwindled (Fig 4L–4N) immediately after 72 h.

**Fig 5:**
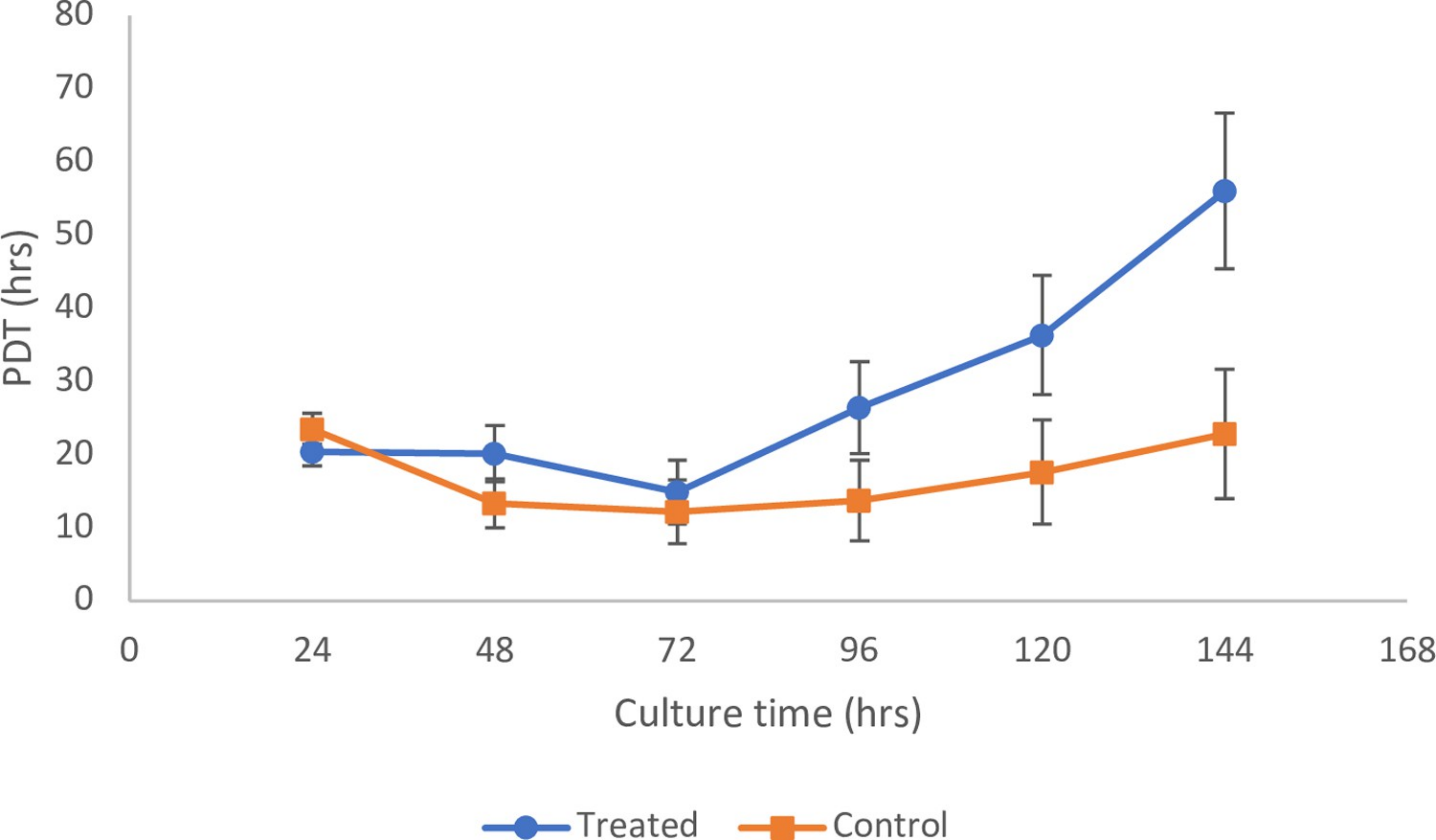
Comparison of population doubling time between control (untreated) and MOL treated hWJ-MSCs at each time point relative to the initial culture time. The results were presented as mean ± SD where n = 3.

*Population doubling time (PDT)*. Based on Fig 5, the PDT showed similar trends for both the treated and control samples, whereby it decreased up to 72 h, followed by an increase for the remainder of the culture time. The average PDT throughout the observed time point (144 h) of the untreated hWJ-MSCs (control) was 17.2 ± 5.2 h, whereas for MOL treated hWJ-MSCs was 29.0 ± 5.9 h. This indicates that the MOL treatment slowed down the proliferation of the hWJMSCs. Also, it is worth noting that the average PDT of our control MSCs (passage 2) were in close agreement with a previous study on 2^nd^ generation hWJMSCs that had a PDT of 17.7 ± 0.5 h [41]. Furthermore, the PDT in our study was lower than a study by Bharti *et al*. [42] on early passage hWJ-MSCs where they noted a PDT range of 49.6 and 51.5 h. This could be due to our study’s low initial seeding density (1500 cells/cm^2^) compared to theirs at 5000 cells/cm^2^. It has been shown that there is an inverse relationship between seeding density and population doublings [43], where seeding densities of hWJ-MSCs below 5000 cells/cm^2^ all exhibited higher rates of population doubling.

#### The differentially expressed genes (DEGs) induced by MOL

Using a cut-off fold change of log_2_ > 1 and p < 0.05, a total of 650 differentially expressed genes (DEGs) were identified where 451 genes were up-regulated while 199 genes were down-regulated. The log_2_ fold change values for the up-regulated DEGs ranged between 1.326 and 7.451 and between -1.343 and -8.670 for the downregulated DEGs. The top 20 significant DEGs are shown in Table 2.

**Table 2:** Top 10 significant DEGs (up and down-regulated) when comparing the gene expression of MOL-treated hWJMSCs and untreated hWJMSCs (control). Positive log_2_ fold change value indicates greater expression on MOL-treated hWJMSCs.

No.	Ensembl gene ID	Gene name	Log_2_ fold change	p-value
Up-regulated				
1.	ENSG00000231628	AL133406.2	7.451203	0.000165
2.	ENSG00000267141	AC012615.4	7.230184	0.000391
3.	ENSG00000258515	AL355075.2	7.148287	0.000530
4.	ENSG00000207721	MIR186	7.06146	0.001006
5.	ENSG00000150722	PPP1R1C	7.06146	0.001006
6.	ENSG00000279347	AC021945.1	7.06146	0.001006
7.	ENSG00000278467	AC138393.3	6.96907	0.001409
8.	ENSG00000248115	AC023154.1	6.76439	0.002864
9.	ENSG00000271754	AL355802.2	6.76439	0.002864
10.	ENSG00000165181	C9orf84	6.76439	0.002864
Down-regulated				
1.	ENSG00000280800	FP671120.6	-8.67026	1.06E-17
2.	ENSG00000066248	NGEF	-7.65869	7.45E-05
3.	ENSG00000233654	AC108047.1	-6.96271	0.001006
4.	ENSG00000196366	C9orf163	-6.87037	0.001409
5.	ENSG00000281181	FP236383.3	-6.87037	0.001409
6.	ENSG00000227811	INKA2-AS1	-6.66582	0.002864
7.	ENSG00000176381	PRR18	-6.42739	0.006149
8.	ENSG00000269038	AP001462.1	-6.42739	0.006149
9.	ENSG00000088002	SULT2B1	-6.42739	0.006149
10.	ENSG00000163009	C2orf48	-6.1416	0.014079

AL133406.2 was the most significantly up-regulated gene at 7.451-fold, while FP671120.3 was the most significantly down-regulated gene at -8.670-fold in response to the MOL treatment in hWJMSCs. These genes are classified as novel transcripts. Therefore BLASTn was performed in NCBI to find the nearest similar sequence. Firstly, the FASTA format for the genes was obtained from Ensembl, and then the BLASTn search was done using the standard database [nucelotiode collection (nr/nt)] for *Homo sapiens* (taxid:9606). The search criteria were set to ’Highly similar sequences (megablast)’. The top match for AL133406.2 was the NG_011472.1 (Query cover = 100%; E-value = 0.0; Percentage similarity = 100%) gene, and the top match for FP671120.3 was the KY962518.1 (Query cover = 100%; E-value = 0.0; Percentage similarity = 99.95%) gene. NG_011472.1 is the prolyl endopeptidase (PREP) gene, where the peptidase encoded by this gene can cleave short peptides at the C-side of an internal proline and plays a role in several apoptotic pathways [44]. KY962518.1 is a gene that encodes for human 18S rRNA (Ensembl release 103) [45]. Several DEGs identified were known to be involved in several cancers, these include the microRNA MIR-186 and the PPP1R1C genes, both of which were up-regulated in our study, and NGEF, C9orf163, INKA2-AS1 and SULT2B1 which were found to be downregulated [46–51].

#### GSEA analysis of DEGs

**Fig 6:**
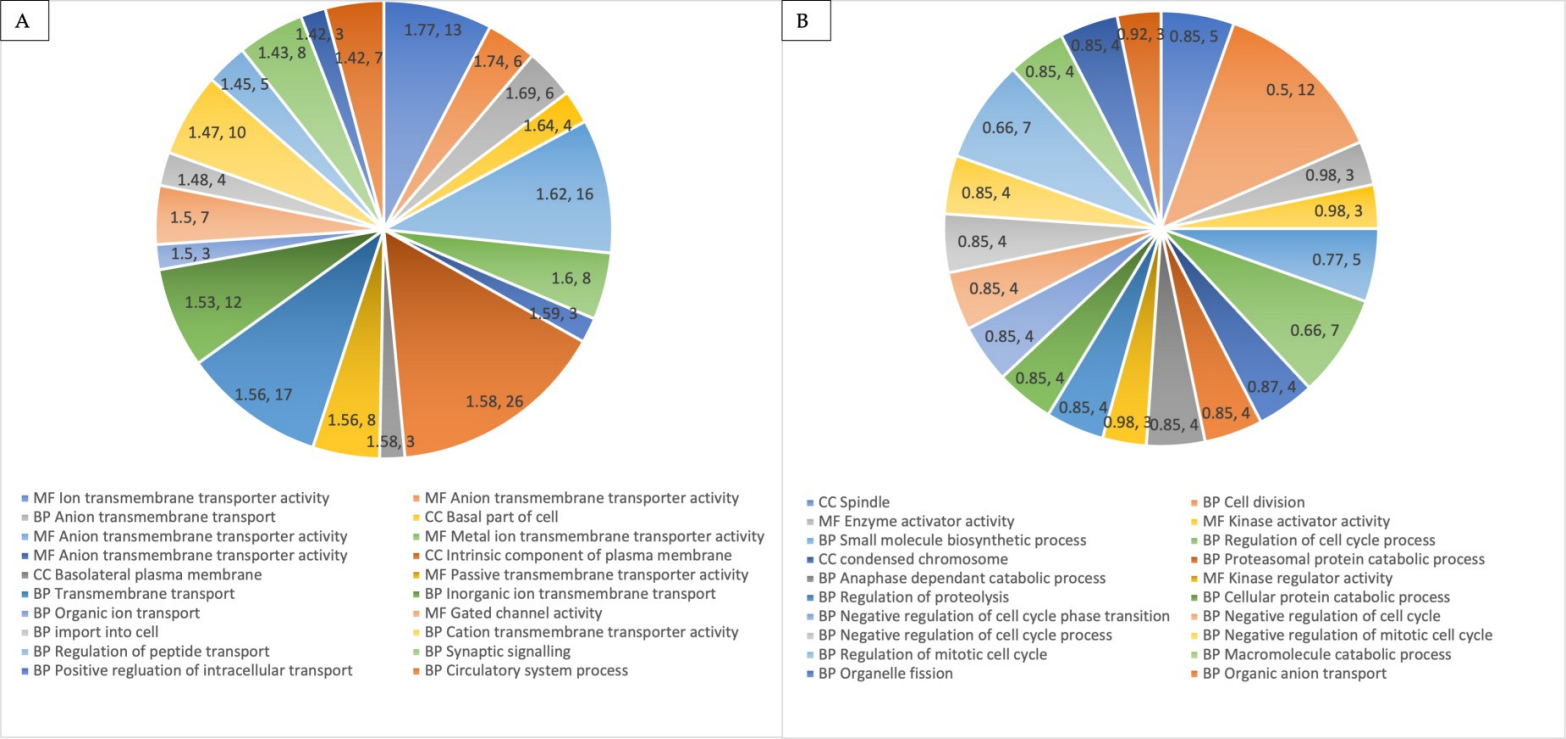
Top 20 significantly overrepresented gene ontology, GO terms in up-regulated (A) and down-regulated (B) DEGs The numbers in the chart represent the enrichment score as calculated by GSEA and the size (number of genes) in each term. BP = Biological process; MF = Molecular function; CC = Cellular component.

To better understand the biological processes in our hWJMSCs upon the MOL extract treatment, we compared its DEGs to gene sets belonging to the entries in the Gene Ontology database using the open-platform tool known as gene set enrichment analysis (GSEA). The results (Fig 6) showed that the up-regulated genes significantly (p < 0.05) enriched 16 gene sets, whereas the down-regulated genes significantly enriched 87 gene sets at p < 0.05. The up-regulated DEGs were mainly found to enrich GO terms related to transmembrane transporter activity, whereas the down-regulated DEGs enriched functions related to the cell cycle regulation. The top 10 most significant GO terms for the up-regulated and down-regulated genes are shown in Table 3. Regarding KEGG analysis, the results showed that the up-regulated genes did not significantly (p > 0.05) enrich any pathways, however, the down-regulated genes significantly enriched (p < 0.05) 3 pathways. The top enriched KEGG pathways for the down-regulated genes were cell cycle, oocyte meiosis, and endocytosis.

**Table 3:** Top 10 significant GO terms (up and down-regulated) when comparing the gene expression of MOL-treated hWJMSCs and untreated hWJMSCs (control).

No.	Ensembl gene ID	Gene ontology	p-value
Up-regulated			
1.	Ion Transmembrane Transporter Activity	MF	0.004
2.	Anion Transmembrane Transporter Activity	MF	0.004
3.	Anion Transmembrane Transport	BP	0.007
4.	Basal Part of Cell	CC	0.005
5.	Transporter Activity	MF	0.015
6.	Metal Ion Transmembrane Transporter Activity	MF	0.021
7.	Ligand Gated Ion Channel Activity	MF	0.010
8.	Intrinsic Component of Plasma Membrane	CC	0.006
9.	Basolateral Plasma Membrane	CC	0.006
10.	Passive Transmembrane Transporter Activity	MF	0.023
Down-regulated			
1.	Spindle	CC	< 0.001
2.	Cell Division	BP	0.009
3.	Enzyme Activator Activity	MF	< 0.001
4.	Kinase Activator Activity	MF	< 0.001
5.	Small Molecule Biosynthetic Process	BP	0.004
6.	Regulation of Cell Cycle Process	BP	< 0.001
7.	Condensed Chromosome	CC	< 0.001
8.	Proteasomal Protein Catabolic Process	BP	< 0.001
9.	Anaphase Promoting Complex Dependant Catabolic Process	BP	< 0.001
10.	Kinase Regulator Activity	MF	< 0.001

BP = Biological process; MF = Molecular function; CC = Cellular component.

#### Canonical pathway analysis

**Fig 7:**
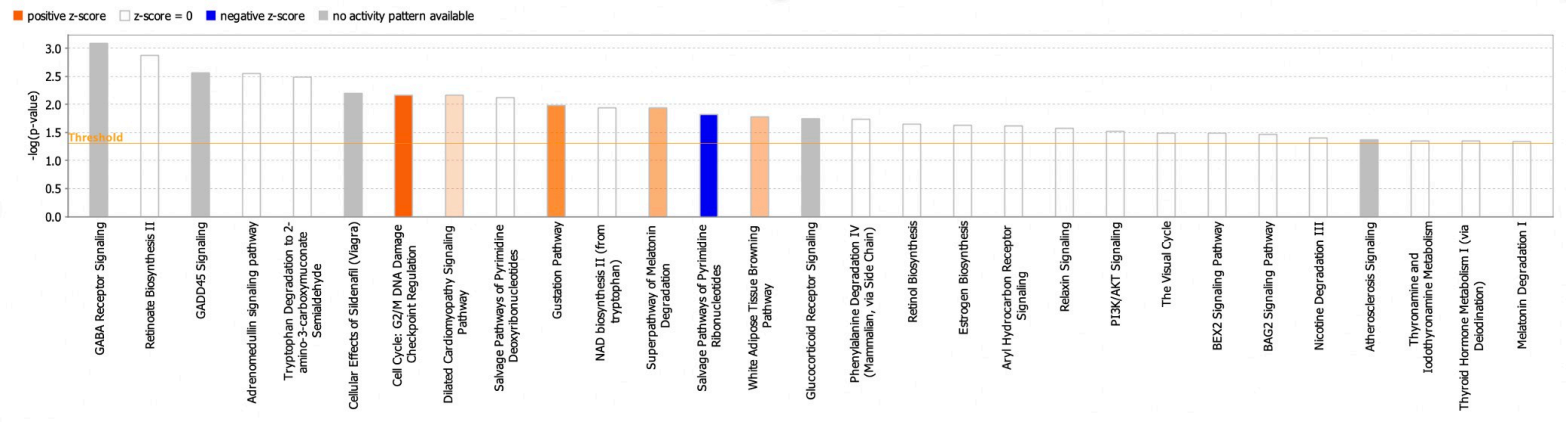
The canonical pathways identified by IPA are based on the DEGs in MOL-treated hWJMSCs. The filter criteria employed is −log (p-value) > 1.3, and the orange line denotes the p-value threshold. Significance was calculated by IPA using Fischer’s exact test, where taller bars indicate increased significance. The bars are coloured according to IPA’s predicted activity status for that particular pathway using the z-score where orange shows activation (z > 2), blue shows inhibition (z < -2) and grey means no available activity pattern.

**Fig 8:**
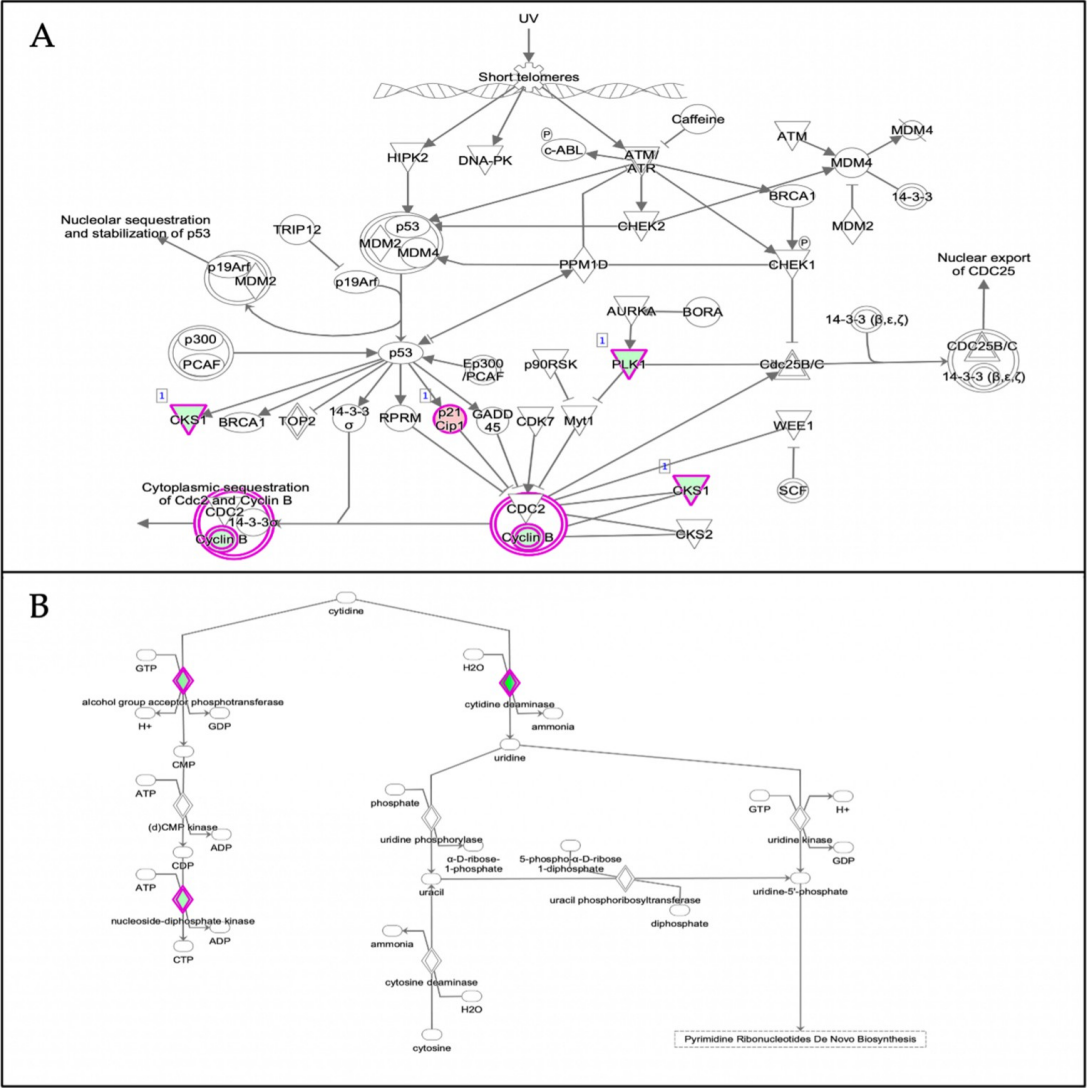
Depiction of the two main canonical pathway webs of a network as predicted by IPA based on the DEGs in MOL-treated hWJMSCs based on the filter criteria of p < 0.05 and z-score greater than 2 or smaller than– 2. A) Cell cycle: G2/M DNA damage checkpoint regulation (z-score = 2.000); B) Salvage pathways of pyrimidine ribonucleotides (z-score = - 2.236). Nodes represent molecules in a pathway, while the relationship between the nodes is represented by lines (edge). The reddish-purple border depicts significantly changed molecules where green colour represents downregulation and red colour means upregulation; white shapes represent molecules the pathway that was not found in our dataset. Nodes are displayed using different shapes that represent the various functional class of a gene product. For more information on the shapes and relationship type, please refer to http://qiagen.force.com/KnowledgeBase/articles/Basic_Technical_Q_A/Legend.

Canonical pathways in the Ingenuity Pathway Analysis (IPA) software are well-defined biochemical cascades that transduce specific functional biological consequences [52]. The top enriched canonical pathways identified by applying the −log (p-value) > 1.3 thresholds are shown in Fig 7. From Fig 7, the top 5 canonical pathways were GABA receptor signalling, retinoate biosynthesis II, GADD45 signalling, adrenomedullin signalling pathway and tryptophan degradation 2-amino-3-carboxymuconate semialdehyde. However, only 2 pathways passed the additional filter criteria based on the z-score, which is the ’Cell cycle: G2/M DNA damage checkpoint regulation (z-score = 2.000)’ pathway shown as a dark orange bar and the ’Salvage pathway of pyrimidine ribonucleotides (z-score = 2.236) shown as a dark blue bar. Fig 8 below depicts the IPA predicted web of networks for the above pathways, which passed the z-score criteria.

#### Upstream regulator analysis

**Fig 9:**
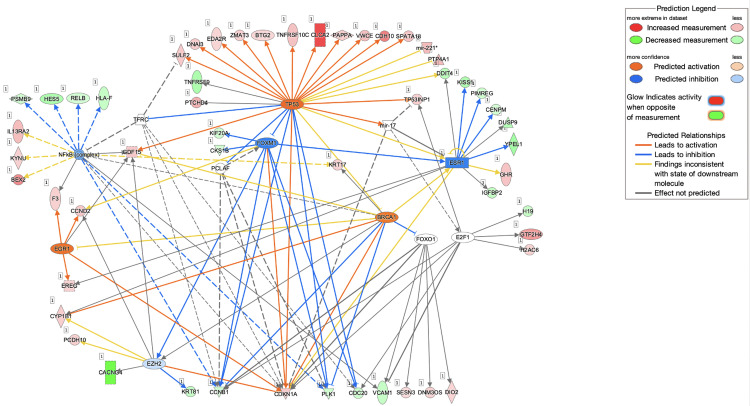
A mechanistic network showing the relationship between TP53 and several other sets of connected upstream regulators could elicit the expression of the DEGs observed in MOL-treated hWJMSCs. Nodes represent gene products and red coloured nodes indicate up-regulated genes and green coloured nodes indicate down-regulated genes; Edges represent direct (solid line) and indirect (dashed line) interactions between molecules based on information present within the Ingenuity knowledge base; Node shapes are related to functional classes where vertical rectangles are for G-protein coupled receptors, horizontal rectangles for ligand-dependent nuclear receptor, squares for cytokines, triangles for phosphatases, inverted triangles for kinases, vertical diamonds for enzymes, horizontal diamonds for peptidase, vertical ellipses for transmembrane receptors, horizontal ellipses for transmembrane regulators and circles for complex/other.

The upstream regulator analysis is a novel function in IPA that helps relate the DEG pattern in a dataset to potential upstream regulators like transcription factors (TFs) or genes that have been experimentally observed [31]. Table 4 depicts the IPA predicted upstream regulators and their activation pattern in our MOL-treated hWJMSCs. Fig 9 depicts our study’s predicted topmost activated upstream regulator, TP53 with other regulators and the DEGs.

**Table 4:** The upstream regulators predicted by IPA are based on the DEGs in MOL-treated hWJMSCs.

Upstream Regulator	Molecule Type	Predicted Activation	Activation z-score	p-value
TP53	Transcription regulator	Activated	3.373	6.11E-04
BTK	Kinase	Activated	2.236	1.01E-03
IGFBP2	Other	Activated	2.217	3.14E-04
TLR7/8	Group	Activated	2	2.97E-03
ANLN	Other	Inhibited	-2.828	7.54E-07
AURK	Group	Inhibited	-2.813	7.54E-07
MITF	Transcription regulator	Inhibited	-2.449	8.82E-03
HLX	Transcription regulator	Inhibited	-2.219	2.25E-05
E2F3	Transcription regulator	Inhibited	-2.219	7.15E-03
CKAP2L	Other	Inhibited	-2	8.94E-03
CBX7	Other	Inhibited	-2	6.59E-05
RABL6	Other	Inhibited	-2	1.88E-02

The prediction of the topmost activated regulator was employed by IPA based on the criteria of p-value < 0.05 and z-score > 2. IPA predicted the activation of this master regulator based on the up-regulation and down-regulation pattern of genes in the treated hWJMSCs related to TP53. The up-regulated genes in our dataset that corresponded to the activation of TP53 were: SPATA18, VWCE, ESR1, TNFRSF10C, ZMAT3, CLCA2, SUL2, DNAI3, PAPPA, TP53INP1, BTG2, CHD10, CDKN1A, GDF15 and EDA2R, whereas down-regulated genes were: CDC20, CCNB1 and PLK1. Conversely, we found that ANLN was the most inhibited upstream regulator with a p-value of 7.54E-07 and a z-score of -2.828. ANLN encodes for an actin-binding protein and plays an important role in cell growth, migration, and cytokinesis. Besides that, IPA also predicted CBX7, CKAP2L and RABL6 (a member of the RAS oncogene family) among other top upstream regulators, with an activation z-score of– 2.000, suggesting their inhibition in both the MSCs.

#### Disease analysis using IPA

**Fig 10:**
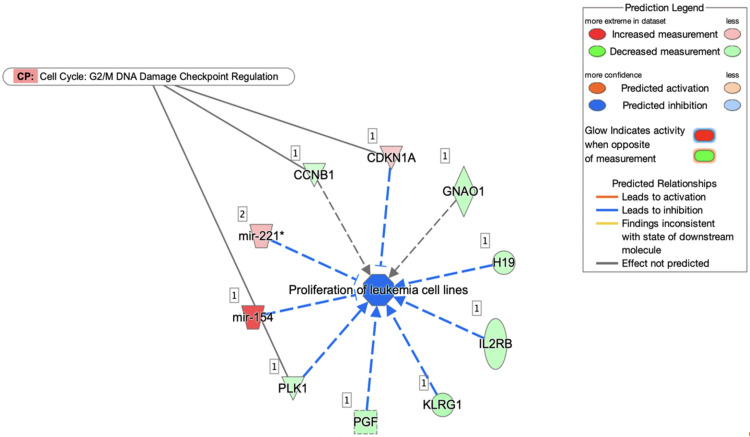
The top-scoring disease network (proliferation of leukaemia cell lines) for MOL-treated hWJMSCs.

From our IPA analysis, only one disease was found to be significantly activated based on the criteria of p-value < 0.05 and z-score > 2 as shown in Fig 10. There were 8 out of 10 genes in our dataset with measurement direction consistent with the decrease in proliferation of leukaemia cell lines. The genes involved are H19, CDKN1A, PGF, IL2RB, KLRG1, mir-154, mir-221 and PLK1.

## Discussion

The UHPLC-QTOF/MS technology has been proposed as a highly accurate and sensitive platform to analyse many different metabolites in parallel [53]. From Table 1, we noticed various phytochemicals in MOL like phenolic acids and flavonoids using this method, consistent with previous reports [54–57]. Most authors have shown that flavonoids like quercetin, apigenin and kaempferol were the predominant phytochemical group in MOL where they usually exist in glycosylated derivatives or linked to sugar moieties [58]. Our results agreed with this as most of the identified compounds existed in their derivative forms, for instance, apigenin-6-C-glucosylglucoside, quercetin-3-O-(6"-O-acetyl)-β-D-glucopyranoside and kaempferol-3-O-β-D-glucopyranoside. Most of the identified compounds like apigenin, luteolin, quercetin, kaempferol and chlorogenic acid are well-known for their high antioxidant, anti-cancer, anti-inflammatory, anti-diabetic and antiviral properties [12, 59–65].

Studies have shown that both isolated compounds and crude extracts of *M*. *oleifera* possess apoptotic activities, especially cancer cells [66]. In particular, apigenin’s effects are well studied. A previous study [67] indicated that it could eliminate cancer stem cells via the overexpression of the antitumour protein, p53. In MSCs, apigenin treatment was shown to cause amplified apoptosis due to an increase in Bad/Bcl-2 and caspase-3 activation, mediated through the suppression of VDR expression [68]. Our study revealed that apigenin was the top compound in our dried MOL extract, and this could have contributed to a similar pattern of amplified cell death based on the cell viability results and morphological analysis in our MOL treated hWJMSCs when compared to the control cells.

To gain better insight into the effects of MOL on the hWJMSCs, RNA-sequencing analysis was performed. We demonstrated that the MOL extract’s cytotoxicity on the cells probably acts through cell cycle arrest or apoptotic pathways. This can be seen from the DEG results (Table 2), where we identified several genes implicated in the apoptotic pathways. The topmost up-regulated gene is prolyl endopeptidase (PREP). PREP plays a vital role in generating the tetrapeptide Ac-SDKP (N-acetyl-seryl-aspartyl-lysyl-proline), inhibiting proliferation by inducing apoptosis in various cell lines [69, 70]. Besides that, we also noticed that genes such as PPP1R1C and microRNAs like MIR186 were significantly up-regulated in the treated hWJMSCs. The PPP1R1C (protein phosphatase 1 regulatory inhibitor subunit 1C) gene possesses antiproliferative and tumour-suppressive functions [71, 72]. MIR186’s involvement in reduced cell growth and apoptotic processes has also been studied, where it binds to and thus decreases the expression of p21-activated kinase 7 (PAK7), which leads to inhibition of proliferation apoptosis, or by repressing the X-linked inhibitor of apoptosis (XIAP) and FOXO1 [73, 74]. On the other hand, genes SULT2B1 were noted to be down-regulated in our MOL-treated hWJMSCs. It is a member of sulfotransferase that catalyses the sulphate conjugation of various hormones and drugs [75]. The inhibition of SULT2B1 hindered cell growth and cell cycle arrests, especially in cancer cells [76, 77]. Therefore, the expression patterns of these genes alongside others in our study could be the reason for the reduced cell growth of hWJMSCs observed in our study.

This is further evident in the Gene Set Enrichment Analysis (GSEA). The gene ontology (GO) results (Table 3) identified the most up-regulated and down-regulated pathways related to transmembrane transporter activity and cell cycle regulation, respectively. Many genes belonging to the solute carrier (SLC) superfamily were noted to have contributed to the upregulation of the pathways associated with transporter activities. SLCs are one of the largest family of transport proteins in mammals and is often up-regulated in response to stresses [78] Therefore, their up-regulation in our study could mean that the MOL treatment-induced stress to the hWJMSCs. Regarding the cell cycle regulation related pathways, several genes like CCNB1, PLK1 and CDC20 were present. CCNB1, also known as cyclin B1, is a crucial component of the cyclin-dependant kinases that regulate division and a cell’s progression through the cell cycle phases. PLK1 codes for serine/threonine-protein kinase, which performs important functions throughout the M phase of a cell cycle, including regulating centrosome maturation and spindle assembly. CDC20 is a regulatory protein that is required for nuclear movement before anaphase and chromosome separation. The downregulation of these genes, which subsequently contributed to the enrichment (downregulation) of cell cycle-related KEGG pathways, suggests that the hWJMSCs proliferation was inhibited by the MOL treatment via activating signalling pathways that promoted cell cycle arrest and negative cell division processes. Up to this point, it is also interesting to note that most of the GO and KEGG terms based on the DEGs like CCNB1, PLK1 and CDC20 are closely related to anti-cancer patterns, which may point to the anti-cancer potential of our MOL extract preparation [79, 80].

The IPA analysis showed several significantly enriched pathways like the GABA receptor signalling and GADD45 signalling pathways. Gamma-aminobutyric acid (GABA) is a major inhibitory neurotransmitter associated with tumour suppressive activities [81, 82]. The growth arrest and DNA damage 45 (GADD45) pathways have been shown to regulate the cell cycle, apoptosis, and DNA repair under various stress stimuli [83, 84]. Using the z-score as an additional filter criteria, the ’Cell cycle: G2/M DNA damage checkpoint regulation (z-score = 2.000)’ pathway and the ’Salvage pathway of pyrimidine ribonucleotides were predicted to be activated and inhibited, respectively. Fig 6 shows the interaction of the genes involved in these pathways. Regarding the ’cell cycle: G2/M DNA damage checkpoint regulation’ pathway (Fig 6A), it is shown that the upregulation of p21 is closely related to the downregulation of cyclin B1 (CCNB1). The CDKN1A gene, which was up-regulated in our MOL-treated hWJMSCs encodes the p21 protein. This protein was previously revealed to play a major role in inducing cells’ G2/M cell cycle arrest by inhibiting the mitotic kinase CCNB1, and its loss is closely associated with prolonged mitosis, which could lead to genomic instability in cells [85–87]. A recent report also revealed that MOL treatment downregulated CCNB1, which contributed to the G2/M cell cycle arrest in Hela cells that inhibited its proliferation [88]. Our results point to a consistent pattern with these studies, which again indicates that the inhibited proliferation of hWJMSCs was most likely due to the MOL treatment inducing cell cycle arrest.

In addition, IPA predicted several upstream regulators that might have contributed to the expression pattern in our study. Among the activated regulators, TP53 was shown to be the topmost activated. Fig 7 depicts the relationship of TP53, and other plausible upstream regulators predicted by IPA, like the NFkB complex, ESR1 and FOXM1 which possibly influenced the expression pattern of the DEGs (TNFRSF9, PTCHD4, ZMAT3, GDF15, SULF2, DNAI3, PAPPA, CDC20, TP53INP1, EDA2R, SPATA18, BTG2, VWCE, CDH10, CDKN1A, CLCA2, ESR1, TNFRSF10C, CCNB1, PLK1, DDIT4, PTP4A1, mir-221) in our MOL-treated hWJMSCs. TP53 encodes for the protein p53, which plays a major role in inducing cell cycle arrest, apoptosis, senescence, DNA repair, or changes in the metabolism of cells. Upon a cell’s exposure to stresses, p53 can lead to a transient arrest in G1 for DNA repair, or it can induce elimination of the stressed or abnormally proliferating cells to mediate tumour suppression [89]. The predicted activation of TP53 in our study could probably help us conclude that the hWJMSCs reacted to stress upon the MOL treatment, as shown in previous reports where p53 inhibited various cell types’ growth and division based on intracellular signalling events caused by stress signals [90]. A study involving MOL showed that their MOL treatment on MCF7 cells resulted in an upregulation of TP53, which contributed to apoptosis [91]. IPA also predicted the significant inhibition of several upstream regulators like CBX7, CKAP2L and RABL6. CBX7 is a member of the polycomb group (PcG) family of proteins and plays an important role in cell proliferation. The CKAP2L gene encodes for a protein that is a major component of centrosomes and plays a crucial role in cell division [92]. RABL6 (also called RBEL1) is a Rab-like GTPase involved in cell growth and survival [93]. The predicted inhibition of these regulators shows consistency with the earlier suggestions that our MOL treatment resulted in negative cell cycle regulation, leading to proliferation inhibition of the hWJMSCs.

Lastly, based on the DEGs in our MOL-treated hWJMSCs, IPA predicted the inhibition of the disease’ proliferation of leukaemia cell lines. From Fig 8, the microRNA known as mir-154 was noticed to have been significantly overexpressed, which helped to contribute to the inhibition of this pathway. Previous reports have also shown that the significant up-regulation of mir-154 resulted in the inhibition of various types of cancers [94, 95]. Another interesting gene that was shown to contribute to this disease inhibition is the CDKN1A gene. As mentioned before, it encodes for p21, a pivotal cell cycle regulator that induces cells’ G2/M cell cycle arrest. CDKN1A is often down-regulated in human cancer cells and its up-regulated expression in our study could explain the IPA’s disease inhibition prediction [85, 96]. Apigenin, a major compound identified in our MOL has been previously reported to have contributed to the inhibition of several different types of cancers via the overexpression of CDKN1A [97, 98]. Furthermore, this IPA prediction is consistent with previous findings that showed *Moringa* was effective against various leukaemia cell lines [99, 100].

## Conclusion

These results were interpreted within the context of the limitations of this study. In summary, our study is the first to show the cytotoxic effects of MOL on hWJMSCs, which resulted in its inhibited proliferation via genes closely associated with negative cell cycle regulation and cell cycle arrest. Further investigations can be conducted using this study as a reference, such as different MOL preparation methods, time points or concentrations. This represents a promising direction of study because a) patients undergoing cell therapies are already known to take antioxidant supplements such as those derived from MOL for possible benefits like anti-inflammation, b) experts have estimated that the global market for MOL-related products can reach USD 9.4 billion by the year 2027 [101] and c) we can test the MOL extract on cancer cell lines due to the strong anti-cancer gene expression noticed in our study. The research exploration from this point forward could contribute to more evidence on MOL’s potential as a high-commodity product in medicine.

## Supporting information

S1 Table Full list of phytocompounds identified using UHPLC-QToF/MS. (XLSX)

S2 Table List of all the DEGs when comparing the gene expression of MOL-treated hWJMSCs and untreated hWJMSCs (control). (XLSX)

S3 Table Full list of the upregulated and downregulated GO and KEGG terms when comparing the gene expression of MOL-treated hWJMSCs and untreated hWJMSCs (control). (XLSX)
